# Preoperative estimation of the prognosis of invasive breast cancer, based on ultrasound size, core biopsy grade and percutaneous axillary lymph node biopsy

**DOI:** 10.1186/bcr2968

**Published:** 2011-11-04

**Authors:** O Elseedawy, C Purdie, L Jordan, S Vinnicombe, P Whelehan, A Thompson, A Evans

**Affiliations:** 1 Ninewells Medical School, Dundee, UK

## Introduction

Assessment of the prognosis of invasive breast cancer prior to surgical resection may influence patient management. The aim of this study was to calculate the Nottingham Prognostic Index (NPI) from preoperative information and compare this with the NPI generated by the surgical pathology findings.

## Methods

The preoperative Nottingham Prognostic Index (PNPI) was calculated in 46 consecutive women undergoing primary surgery for invasive breast cancer. The parameters used were imaging size (normally by ultrasound), tumour grade on core biopsy, and axillary lymph node assessment including core biopsy where appropriate. Values were divided into good (<3.41), moderate 1 (3.41 to 4.4), moderate 2 (4.41 to 5.4) and poor (>5.4) prognostic groups. Intraclass correlation coefficients (ICC) were calculated to compare the PNPI with the postoperative NPI.

## Results

Comparison of the PNPI and NPI gave an ICC of 0.68 (95% CI = 0.48 to 0.81), indicating fair to good agreement. In 39 of 46 women (85%), the PNPI was within one point of the NPI and in 34 (74%) it was within 0.5. Thirty women (65%) were assigned to the correct NPI group by the PNPI. Twelve (26%) were assigned to the adjacent NPI group. In 14 women the NPI group was worse than the PNPI, and in two it was better because the tumours were downgraded at postoperative pathology.

## Conclusion

Preoperative estimation of the NPI approximates to the definitive NPI in the majority of women studied, and could therefore be used to guide systemic treatment decisions preoperatively.

